# Insecticide resistance status of *Anopheles gambiae s.s* population from M’Bé: a WHOPES-labelled experimental hut station, 10 years after the political crisis in Côte d’Ivoire

**DOI:** 10.1186/1475-2875-12-151

**Published:** 2013-05-04

**Authors:** Alphonsine A Koffi, Ludovic P Ahoua Alou, Maurice A Adja, Fabrice Chandre, Cédric Pennetier

**Affiliations:** 1 Institut Pierre Richet (IPR), Abidjan, BP 47, Côte d’Ivoire; 2 Laboratoire de Zoologie et Biologie Animale, Université Felix Houphouët-Boigny de Cocody, Abidjan, 22 BP 582, Côte d’Ivoire; 3 Institut de Recherche pour le Développement (IRD)/UMR224 MiVEGEC, Montpellier, 34394, France; 4 Centre de Recherche Entomologique de Cotonou, Cotonou, 06 BP 2604, Bénin

**Keywords:** Insecticide-resistance, *Anopheles gambiae*, M’Bé, Côte d’Ivoire

## Abstract

**Background:**

An experimental hut station built at M’Bé in 1998 was used for many years for the evaluation of insecticidal product for public health until the civil war broke out in 2002. Breeding sites of mosquitoes and selection pressure in the area were maintained by local farming practices and the West African Rice Development Association (WARDA, actually AfricaRice) in a large rice growing area. Ten years after the crisis, bioassays, molecular and biochemical analyses were conducted to update the resistance status and study the evolution of resistance mechanisms of *Anopheles gambiae s.s* population.

**Methods:**

*Anopheles gambiae s.s* larvae from M’Bé were collected in breeding sites and reared until emergence. Resistance status of this population to conventional insecticides was assessed using WHO bioassay test kits for adult mosquitoes, with 10 insecticides belonging to pyrethroids, pseudo-pyrethroid, organochlorides, carbamates and organophosphates with and without the inhibitor piperonyl butoxyde (PBO). Molecular and biochemical assays were carried out to identify the L1014F *kdr*, L1014S *kdr* and *ace-1*^*R*^ alleles in individual mosquitoes and to detect potential increase in mixed function oxidases (MFO) level, non-specific esterases (NSE) and glutathione S-transferases (GST) activities.

**Results and discussion:**

*Anopheles gambiae s.s* from M’Bé exerted high resistance levels to organochlorides, pyrethroids, and carbamates. Mortalities ranged from 3% to 21% for organochlorides, from 50% to 75% for pyrethroids, 34% for etofenprox, the pseudo-pyrethroid, and from 7% to 80% for carbamates. Tolerance to organophosphates was observed with mortalities ranging from 95% to 98%. Bioassays run with a pre-exposition of mosquitoes to PBO induced very high levels of mortalities compared to the bioassays without PBO, suggesting that the resistance to pyrethroid and carbamate relied largely on detoxifying enzymes’ activities. The L1014F *kdr* allelic frequency was 0.33 in 2012 compared to 0.05 before the crisis in 2002. Neither the L1014S *kdr* nor *ace-1*^*R*^ mutations were detected. An increased activity of NSE and level of MFO was found relative to the reference strain Kisumu. This was the first evidence of metabolic resistance based resistance in *An. gambiae s.s* from M’Bé.

**Conclusion:**

The *An. gambiae s.s* population showed very high resistance to organochlorides, pyrethroids and carbamates. This resistance level relied largely on two major types of resistance: metabolic and target-site mutation. This multifactorial resistance offers a unique opportunity to evaluate the impact of both mechanisms and their interaction with the vector control tools currently used or in development.

## Background

The scaling-up of long-lasting insecticidal nets (LLINs) and to some extent indoor residual spraying (IRS) is the cornerstone element of international strategies to control malaria transmission [1]. Four classes of chemical insecticides are the mainstay of vector control programmes [2], but pyrethroids are the only class of insecticide currently recommended for use on LLINs because of their irritant and fast-acting properties and their safety for humans [3]. Under the selective pressure of insecticides massively used in agriculture [4,5] and also in public health programmes [6,7], pyrethroid resistance has become widespread among *Anopheles gambiae s.l* in sub-Saharan Africa [8-10]. Even in four insecticide classes available for IRS, resistance has been reported for all of them in some populations of *Anopheles gambiae s.s*[11]. Thus the arsenal for managing resistance and providing sustainable vector control with existing chemicals is becoming seriously limited.

Until alternative chemicals arise, manufacturers, national and international authorities bring their experiences together to build new strategies to manage insecticide resistance issues [12]. In this process the most advanced strategy is to combine two chemicals with different modes of action into one LLIN against malaria pyrethroid-resistant vectors [13]. Since a decade, few combinations are under evaluation for resistance management: pyrethroids and organophosphates [14], repellents and organophosphates [15,16], pyrrole insecticide and pyrethroid [17], pyrethroid and the synergist piperonyl butoxyde (PBO) [18-21]. The only combinations that has been manufactured into LLINs and submitted to WHO evaluation process are mosaic or mixture of PBO and pyrethroids (deltamethrin or permethrin).

The World Health Organization Pesticide Evaluation Scheme (WHOPES) reviews and makes recommendations on new pesticide technologies for public health programmes, such as LLINs or IRS. The WHOPES testing and evaluation process is divided into four phases: Phase I: the efficacy is investigated under laboratory conditions against standard (susceptible or insecticide resistant) mosquito strains; Phase II: the efficacy is investigated against wild vector populations in standardized field conditions (experimental huts); Phase III is a review of overall performance in the field at village scale; and, Phase IV consists in the development of WHO specifications for quality control and international trade.

Experimental huts constitute a crucial step for the WHOPES to assess a number of entomological criteria in different entomological settings [12]. A promising new tool must be effective against most, even all of the *Anopheles* vector populations. Indeed the industrial investment to manufacture massive numbers of LLINs is possible under condition of worldwide use. Given the patchy distribution of insecticide resistance mechanisms evolving in *Anopheles* vectors, their different phenotypic effect and the possible interactions, new products must be evaluated against vector populations bearing different resistance mechanisms before being labelled by the WHOPES.

In Côte d’Ivoire, two experimental stations, M’Bé and Yaokoffikro were built in the early 1990s close to Bouaké in the Bandama department [22]. In M’Bé, *An. gambiae s.s* population was known to be 95% M form, 5% S form. The *kdr-w* mutation was only present in the S form at a frequency of 63% representing only 4% of the whole *An. gambiae* population [23].

Until the political crisis in 2002 the *An. gambiae* population was susceptible to pyrethroids, organophosphates and carbamates. The only phenotypic resistance detected was to the dieldrin (cyclodiene organochlorine) and the fipronil (phenylpyrazole) [22]. In contrast, the *An. gambiae* population of Yaokoffikro was exclusively S form *An. gambiae* bearing the *kdr-w* mutation at a very high frequency 95% [22,23], until the political crisis.

The armed conflict led to a lot of population migration across the country, affecting social organization, economical and agricultural activities. At M’Bé, the rice-growing area of the West African Rice Development Association (WARDA, actually Africa Rice), it is unclear whether the crisis changed the local farming and Africa Rice practices that might have led to a shift in selection pressure. At the neighbouring WHOPES experimental hut station of Yaokoffikro, a recent study assessed the resistance status and showed the maintenance of high resistance to pyrethroids, DDT and carbamates in the *An. gambiae s.s* population, having both metabolic and target site mutation [24]. Ten years after the crisis, bioassays, biochemical and molecular analyses were conducted to update the resistance status in the *An. gambiae s.s* population from M’Bé.

## Methods

### Study area

This study was conducted in M’Bé valley (5.209963° W, and 7. 970241° N) situated 30 km north of Bouaké in the central region of Côte d’Ivoire (Bandama department). M’Bé valley is a rice growing area where the Africa Rice, are conducting experimental field rice trials and where local farmers are also producing rice. The rice paddies are suitable breeding sites for mosquitoes especially for *An. gambiae s.s.*. Experimental huts belonging to the “Institut Pierre Richet (IPR)” built in 1998 served over many years for the evaluation of different insecticides under the auspices of WHOPES [22,25-28]. Mosquito population in this area was composed by *An. gambiae s.s*, *Anopheles funestus*, *Culex sp.* and *Mansonia sp*. *Anopheles gambiae s.s* was mostly M molecular form and less resistant to pyrethroids and DDT bearing the Leu-Phe *kdr* mutation (L1014F *kdr*) at an allelic frequency above 0.05 [8,22,23,25,29,30]. Resistance to carbamates and organophosphates involving acetylcholinesterase insensitive has also been detected [28].

### Mosquito collections and maintenance

During May 2012, larvae of *An. gambiae s.s* were collected in the rice paddies of the M’Bé rice growing area around the experimental field station and reared in IPR insectarium until emergence. The *An. gambiae s.s* Kisumu reference strain, which is free of any detectable resistance mechanisms, served as a susceptible control.

### Insecticide susceptibility tests

Susceptibility bioassays on adult mosquitoes were conducted using WHO test kits [31]. Impregnated papers with diagnostic concentrations of 10 insecticide-active ingredients belonging to different chemical classes were prepared and tested as follows:

Pyrethroids: permethrin (0.75%), deltamethrin (0.05%) and α-cypermethrin 0.05%;

Pseudo-pyrethroid: etofenprox (0.05%);

Organochlorides: DDT (4%) and dieldrin (4%);

Carbamates: carbosulfan (0.4%) and bendiocarb (0.1%);

Organophosphates: fenitrothion (1%) and the pirimiphos-methyl (1%).

Filter papers were impregnated according to WHO specifications by the Centre de Recherche Entomologique de Cotonou (CREC) as described by Chandre *et al.*[32]. WHO tube tests were performed with batches of 25 unfed females of *An. gambiae s.s*, two to three days old (four replicates per concentration). Mosquitoes were exposed to the insecticide-treated papers for 60 min at 25 ± 2°C and 80% relative humidity (RH). The number of mosquitoes knocked down at regular intervals during the exposure period was scored and time to knock down 50% and 95% of the exposed mosquitoes (KDT50) and (KDT95) were determined. After the exposition period, mosquitoes were transferred to the observation tube of the test kit. They were supplied with 10% honey solution and held for 24 h before scoring mortality. Batches exposed to untreated papers were used as negative control.

In order to assess the involvement of detoxifying enzymes in the resistance phenotypes, complementary tests were performed with a 1 h pre-exposition to PBO, an inhibitor of oxidases and esterases. Wild mosquito population was compared to a susceptible reference strain of *An. gambiae s.s* Kisumu. All control survival specimens (including the susceptible reference mosquito) from none exposed to insecticides were stored at −80°C for biochemical analysis. The samples of mosquitoes exposed to different insecticides were kept at −20°C for molecular analysis.

### Identification of sibling species and *Anopheles gambiae s.s* M and S molecular forms

Ribosomal DNA was extracted from individual mosquitoes following Collins *et al.*[33] and used for PCR analysis to determine the species following Scott *et al.*[34] and the M and S molecular forms according to Favia *et al.*[35].

### PCR detection of the L1014F and L1014S *kdr* and *ace-1*^*R*^ mutations

The presence of L1014F and L1014S *kdr* alleles was assessed using hot oligonucleotide ligation assay (HOLA) technique according to the protocol of Lynd *et al.*[36]. The PCR-RFLP diagnostic test was used to detect the presence of G119S mutation (*ace-1* gene) as described by Weill *et al.*[37].

### Biochemical analysis

Biochemical assays were performed to compare the amount of mixed function oxidases (MFO), and the activity levels of non-specific esterases (NSE) for ß and α-naphtyl acetate and glutathione S-transferases (GST) [38] in the wild *An. gambiae s.s.* from M’Bé relative to the susceptible Kisumu strain. Mosquitoes used for the biochemical analysis had not been exposed to any insecticides prior to the assay.

### Data analysis

WHO criteria [39] were adopted to define resistance status of the mosquito populations. When less than 80% mortality was observed the population was considered ‘resistant’; between 80 and 97% mortality the population was considered ‘tolerant’ (or ‘suspected of resistance’ in the literature) and when the mortality was above 97% the population was considered ‘susceptible’. Knockdown data were analyzed using the PoloPlus 1.0 software (LeOra Software). KDT_50_ and KDT_95_ were generated by means of a logtime probit model. The KDT_50_ and KDT_95_ generated were compared with that of the *An. gambiae* Kisumu reference susceptible strain by estimates of KDT_50_ and KDT_95_ ratios (RR).

Biochemical assay data (enzymatic activity per mg protein, levels of MFO, NSE and GST) of Kisumu and M’Bé *An. gambiae s.s* were compared using Mann–Whitney non-parametric *U*-test (Statistica software). Conformity of L1014F and L1014S *kdr* and *ace-1*^*R*^ mutation frequencies with Hardy-Weinberg equilibrium was tested for *An. gambiae s.s* population from M’Bé using the exact probability test [40]. Statistical significance was set at the 5% level.

## Results

### Bioassays

#### Knock-down effect

The knock-down effects of organochlorides and pyrethroids on the *An. gambiae s.s* population from M’Bé compared to Kisumu strain are summarized in Table 1. The median knock-down time (KDT_50_) ranged from 15.4 to 24.7 min and the KDT_95_ ranged from 19.5 to 39.5 min with the reference susceptible strain Kisumu. In contrast, the KDT_50_ ranged from 83.2 to 93.7 and the KDT_95_ ranged from 204.7 to 341.6 with the M’Bé *An. gambiae s.s.* population. None of the M’Bé *An. gambiae s.s* mosquitoes were knocked down within the 60 min of contact with the DDT. These results indicate a strong resistance level illustrated by the resistant ratio (RR_50_ and RR_95_) ranging respectively from 3.8 to 5.1 and from 7.4 to 8.6.

**Table 1:** **Knock-down time of *Anopheles gambiae s.s *from M’Bé exposed to pyrethroids and DDT relative to the reference Kisumu *Anopheles gambiae s.s *strain**

**Insecticide**	**Strain**	**N**	**KdT**_**50 **_**(CI**_**95 **_**)**	**KdT**_**95 **_**(min)**	**RR**_**50**_	**RR**_**95**_
DDT 4%	Kisumu	101	15.4 (12.8-18.5)	19.5 (14.1-27.1)	_	_
	M’Bé	193	NA	NA	NA	NA
***DDT 4% + PBO***	***M’Bé***	***105***	***NA***	***NA***	***NA***	***NA***
Permethrin 0.75%	Kisumu	101	17.3 (16.3-18.4)	23.9 (21.8-28.1)	_	_
	M’Bé	201	83.1 (73.9-99.2)	204.7 (155.7-313.7)	4.8 (4.1-5.6)	8.6 (6.0-12.2)
***Permethrin 0.75% + PBO***	***M’Bé***	***100***	***94.9 (76.2-134.7)***	***469.9 (276.8-1 146.7)***	***5.5 (4.1-7.2)***	***19.6 (9.9-38.5)***
α-cypermethrin 0.05%	Kisumu	99	17.8 (12.9-22.8)	39.1 (29.2-74.1)	_	_
	M’Bé	197	90.3 (75.5-118.9)	341.6 (224.3-673.1)	5.1 (4.2-6.1)	8.7 (5.7-13.3)
***α-cypermethrin 0.05% + PBO***	***M’Bé***	***106***	***28 (26.4-29.6)***	***59.4 (54.3-66.4)***	***1.6 (1.4-1.7)***	***1.5 (1.3-1.8)***
Deltamethrin 0.05%	Kisumu	126	19.6 (16.8-22.5)	37.4 (31.3-50.4)	_	_
	M’Bé	208	83.2 (73.6-98.3)	274.9 (205.5-415.4)	4.2 (3.6-4.9)	7.4 (5.1-10.5)
***Deltamethrin 0.05% + PBO***	***M’Bé***	***103***	***41.4 (37.9-45.7)***	***99.1 (81.7-133.3)***	***2.1 (1.9-2.3)***	***2.6 (2.2-3.2)***
Etofenprox 0.5%	Kisumu	105	24.7 (21.9-27.5)	39.5 (34.4-49.7)	_	_
	M’Bé	200	93.7 (80.7-116.9)	296.5 (210.7-499.4)	3.8 (3.1-4.6)	7.5 (4.9-11.5)
***Etofenprox 0.5% + PBO***	***M’Bé***	***91***	***83.2 (65.7-139.2)***	***265.1 (152.7-989.6)***	***3.4 (2.7-44.2)***	***6.7 (3.9-11.5)***

*CI*: confidence interval; *KDT*: knock-down time; *KDT*_*50*_: time taken for 50% of the test mosquitoes to be knocked down; *KDT*_*95*_: time taken for 95% of the test mosquitoes to be knocked down; *RR*: knock-down time ratio (KDT_50_ of the tested population/KDT_50_ of the susceptible Kisumu strain); *NA*: not available.

#### Mortality

**Figure 1:**
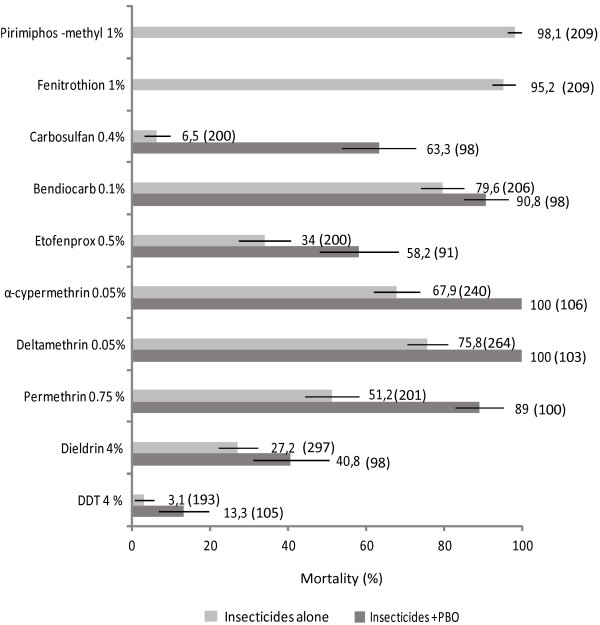
Insecticidal effects of diagnostic concentrations of insecticides (60 min contact in WHO tube tests) with or without a 60 min pre-exposition to PBO.

The mortalities induced by all insecticides on *An. gambiae s.s* M’Bé (with and without a pre-exposition to PBO) are illustrated in Figure 1 (light bars). In the negative control, mortalities were always below 5%. Diagnostic concentrations of all insecticides killed 99 or 100% of *An. gambiae s.s* Kisumu, the susceptible control, confirming the good quality of the impregnated papers.

All organochlorides (DDT and dieldrin), the carbamates (bendiocarb and carbosulfan) and the four pyrethroids (and pseudo-pyrethroids) killed less than 80% of *An. gambiae s.s.* from M’Bé. Based on the WHO criteria, the *An. gambiae s.s* population from M’Bé is considered resistant to all insecticides cited above. It is interesting to note that the strongest resistance levels were observed with DDT (3.1% mortality), dieldrin (21.8% mortality) and the carbosulfan (6.5% mortality). The strongest resistance level observed with a pyrethroid insecticide was with permethrin (50.8% mortality).

The organophosphates fenitrothion and pirimiphos-methyl killed respectively 95.2% and 98.1% of mosquitoes (Figure 1). Based on the WHO criteria, the *An. gambiae s.s* population from M’Bé is considered susceptible to pirimiphos-methyl and tolerant to fenitrothion.

#### Bioassays after a pre-exposition to PBO

When the *An. gambiae s.s.* mosquitoes from M’Bé displayed resistance to an insecticide, WHO bioassays were performed with a 1 h pre-exposition to PBO. After pre-exposure to the PBO α-cypermethrin and deltamethrin induced 100% mortality indicating that resistance of *An. gambiae s.s.* from M’Bé to these pyrethroids relied exclusively on the oxidase and/or esterase activities (Figure 1, dark bars). With permethrin and etofenprox, the mortalities increased respectively from 34% and 51.2% to 63.3% and 89% indicating that the oxidase and esterase activities are largely involved in the resistance to these pyrethroids (or pseudo-pyrethroid) but are not responsible of the whole resistance. The same trend was observed with carbosulfan and bendiocarb (in a lesser extent). The mortalities respectively increased from 6.5% and 79.6% to 63.3% and 90.8% after pre-exposure to PBO. In contrast the PBO did not largely increase the mortalities induced by the organochloride insecticides (3.1% to 13.3%; Khi^2^ = 9.67, p = 0.002 for DDT and 27.3% to 40.8%; Khi^2^ = 3.22, p = 0.074 for dieldrin) indicating that MFO and NSE are not (or slightly involved) responsible for the major part of resistance.

The same trend was observed with α-cypermethrin, deltamethrin with a decrease of both KDT_50_ and KDT_95_ reducing significantly the RR_50_ (respectively from 5.1 and 4.2 to 1.6 and 2.1) and RR_95_ (respectively from 8.8 and 7.4 to 1.5 and 2.6). The decrease of the RR50 and RR95 with etofenprox was not significant (respectively 3.8 and 7.5 to 3.4 and 6.7 with overlapping confidence intervals). Surprisingly whereas the PBO pre-exposition increased the mortality induced by permethrin, it did not decrease the KDT_50_ and KDT_95_.

#### Molecular and biochemical analyses

Among the 226 mosquitoes, three specimens were *An. gambiae s.s* S form, corresponding to 1.3% of the population. Neither *ace-1*^*R*^ mutation nor the *kdr-e* mutation (L1014S) were detected in the M’Bé population sample (Table 2). Concerning the *kdr-w* mutation (L1014F), the genotypes were distributed as follow among the M-form *An. gambiae*: 16 were resistant homozygotes (RR), 114 were heterozygotes (RS), and 93 were susceptible homozygotes (SS). The *kdr-w* frequency was 0.33. The three S-form specimens were respectively SS, RS and RR.

**Table 2:** **Genotype frequencies of the *kdr*, *ace-1 *locus and mean level of NSE, MFO and GST activity in *Anopheles gambiae s.s. *Kisumu and M’Bé**

	**Kisumu**	**M’Bé**
F(*kdr-w*)	-	0.33 (226)
***F(kdr-e)***	***-***	***0.00 (226)***
F(*ace-1*^*R*^)	-	0.00 (226)
***α-Na***	***0.086 ±0.007***^***a ***^***(40)***	***0.155 ±0.012***^***b ***^***(40)***
β-Na	0.084 ±0.007^a^ (40)	0.125 ±0.017^b^ (40)
***MFO***	***0.095 ±0.008***^***a ***^***(38)***	***0.198 ±0.020***^***b ***^***(36)***
GST	0.295 ±0.032^a^ (40)	0.378 ±0.085^a^ (40)

*α*-Na: NSE activity with substrate alpha-naphthyl acetate (μmol *α*-naphthol produced/min/mg protein).

*β*-Na: NSE level with substrate beta-naphthyl acetate (μmol *β*-naphthol produced/min/mg protein).

MFO: MFO level (nmol equivalent unit of cytochrome P450/mg protein).

GST: GST level (nmol GSH conjugated/min/mg protein).

(): Number tested of *Anopheles gambiae s.s.* females.

Different letter superscripts indicates enzyme level significantly higher than with the Kisumu susceptible *An. gambiae s.s*. strain (P < 0.05).

Table 2 shows the mean amount of MFO, and mean activities of NSE and GST of *An. gambiae s.s* from M’Bé relative to the susceptible reference Kisumu strain. The mean NSE respectively α and β-esterases activities (respectively 0.155 and 0.125) and MFO (0.198) level were significantly higher in *An. gambiae s.s* from M’Bé than in the Kisumu specimens (respectively 0.086 and 0.084 for α and β-esterases, and 0.095 for MFO) (P < 0.001). The level of GST activities did not differ significantly between the samples from M’Bé (0.378) than from Kisumu samples (0.295; P > 0.05).

## Discussion

Ten years after the armed conflict in Côte d’Ivoire, the resistance status of the *An. gambiae s.s* population from M’Bé station has been updated in order to have background knowledge to restart the pesticide evaluation activities in the context of the ABC Network. Historically, M’Bé was considered an insecticide-susceptible *An. gambiae s.s* population. Indeed until the political crisis in 2002, the *An. gambiae s.s* population was susceptible to pyrethroids, organophosphates and carbamates. The only phenotypic resistance detected was to dieldrin (organochloride) and fipronil (phenylpyrazole) [22]. In the present study, the M’Bé *An. gambiae s.s* population was resistant to organochlorides, pyrethroids, carbamates and is ‘tolerant’ to organophosphates. Bioassays with a pre-exposition to PBO, an inhibitor of esterases and oxidases, evidenced the strong involvement of these enzyme families in the resistant phenotypes. With the molecular and biochemical assays two types of resistance mechanisms were identified: 1) the *kdr-w* target site mutation with a relatively low frequency (0.33); and, 2) the over-activities of MFO and NSE. In contrast, neither *kdr-e* nor *ace-1*^*R*^ mutations were detected in the M’Bé population. Moreover GST did not display over activity.

These striking results exerted the rapid evolution of the resistance mechanisms among *An. gambiae s.s* populations in such environment. Further investigation must be conducted in order to understand the changes in agricultural practices or socio-economic context that might explain the shift from a susceptible to strongly resistant population. The only well-known agricultural pressure in this area is the treatment of the rice paddies with deltamethrin-based product by AfricaRice (Ahoua Alou, pers comm). The practices of the local farmers are not available yet. This agricultural selective pressure might have been involved in the resistance mechanisms as already showed in several countries [9,41-43]. Moreover the automatic distribution of LLINs to pregnant women and children under five, since 2006, and the implementation of the universal coverage with LLINs launched recently might have added a supplementary selective pressure [44]. Indeed evidences of the selective pressures induced by the massive use of LLINs are more and more documented [7,45].

The distribution of the *kdr-w* genotypes showed that only 7.5% of *An. gambiae s.s* were homozygote resistant. This mutation is well known to be recessive. This suggests that the impact of the *kdr-w* mutation on the phenotypic resistance is relatively low. This was confirmed by the PBO pre-exposure bioassays during which most of the insecticides recovered a high efficacy.

NSE and MFO seem to be responsible for most of the phenotypic resistance in this *An. gambiae s.s* population. The specific genes of NSE and MFO will have to be identified using gene expression or proteomic tools to determine if they correspond to genes already suspected in pyrethroid resistance or if these are new genes [46].

The results showed carbamate resistance in *An. gambiae s.s* population from M’Bé, whilst only a high level of mortality was found with organophosphate. The absence of cross-resistance to carbamates and to organophosphates is confirmed by the absence of the *ace-1* G119S mutation, despite N’Guessan *et al.*[28] reported reduction of the acetylcholinesterase activities in the M’Bé *An. gambiae s.s* population in 2003. In this study N’Guessan *et al.* did not search for the *ace-1*^*R*^ mutation. In the present study, the absence of the *ace-1* G119S mutation in M’Bé *An. gambiae s.s* population associated with the resistance to carbamate strongly supports the involvement of metabolic resistance based on the high activities of NSE or MFO and the significant increase of mortalities using the synergist PBO. The involvement of NSE and MFO at M’Bé in *An. gambiae* M molecular form was also reported in the field experimental station of Pitoa (Cameroon), where greater oxidase and esterase activities were observed in *An. gambiae s.s*[47-49] and where *kdr* and *ace-1*^*R*^ were also absent. In contrast, in Yaokoffikro the field experimental hut station in Côte d’Ivoire, 40 km from M'Bé, greater GST and esterase activities were observed in *An. gambiae s.s* associated with high frequencies of L1014F *kdr* (0.94) and *ace-1*^*R*^ (0.50) mutations, but in this place 100% of mosquitoes belong to the S form [24].

This new results highlight once again the high variability in insecticide resistance patterns and evolution processes driving the resistance mechanisms evolution among malaria vectors. In terms of vector control research and vector control tool development, it appears crucial to take into account these different ecological patterns and evolution processes. The M’Bé *An. gambiae s.s* population is currently one of the rare *An. gambiae s.s* population in west Africa bearing the *kdr-w* mutation at relatively low frequency (0.33). It offers a unique opportunity to deeply study the impact of such metabolic mechanisms on resistance phenotypes. Undergoing program aims to describe and select enzymatic mechanisms involved in resistance phenotype specific to the main insecticide families used for public health (organochlorides, pyrethroids, carbamates and organophosphates).

In the context of alternative vector control tool development, the M’Bé station will allow scientists to study and quantify the benefit to use chemicals combinations or new active ingredient to control *An. gambiae s.s* vectors bearing different mechanisms in the same area. It is especially rare and important to evaluate the efficacy of vector control tools in development against *An. gambiae s.s* bearing metabolic mechanisms without the *kdr-w* mutation.

## Conclusion

In a 10-year period, the *An. gambiae s.s* population of M’Bé area shifted from susceptibility to high resistance to three insecticide families usually used in public health control programmes (organochlorides, pyrethroids, carbamates) except the fourth one (organophosphates). The resistance pattern is unusual and offers an ideal context for further investigation on the interaction and evolution processes of metabolic resistance and *kdr-w* target site mutation. Trials to evaluate their impact on the protective efficacy of malaria control interventions, as well as new tools in development to manage these complex mechanisms, are urgently needed.

## Abbreviations

LLINs: Long-lasting insecticidal nets; IRS: Indoor residual spraying; WHOPES: World Health Organization Pesticide Evaluation Scheme; IPR: Institut Pierre Richet; L1014F kdr: Leucine-phenylalanine knockdown resistance; L1014S kdr: Leucine-serine knock-down resistance; ace-1R: Acetylcholinesterase-1 resistance; ace-1 G119S: G199S mutation in *ace-1*; NSE: Non-specific esterase; MFO: Mixed-function oxidase; GST: Glutathione S-transferase; PCR: Polymerase chain reaction; DDT: Dichloro-diphenyl-trichloroethane; R: Resistant; S: Susceptible; GSH: Reduced form of glutathione; RH: Relative humidity.

## Competing interests

The authors declare that they have no competing interests.

## Authors’ contributions

AAK, FC and CP designed the study. AAK, LPAA and AMA supervised and conducted the field work. LPAA conducted the laboratory work. AAK and LPAA drafted the paper. CP and FC critically revised the manuscript. All authors read and approved the final manuscript.
